# Referee acknowledgement for 2017

**DOI:** 10.1038/s41416-018-0151-5

**Published:** 2018-07-13

## Abstract

In this issue, we publish the names of those who reviewed the manuscripts for us in 2017. The Editor-in-Chief, Subject Editors and everyone involved in publishing *BJC* would like to extend our sincere thanks to the referees for contributing their expertise and time.

Philippe A. Cassier

Lauri A. Aaltonen

Mohamed Abdel-Rahman

Eric O. Aboagye

Thomas Abrams

Alessio Accardi

Marc G. Achen

Anya Adair

Peter Adams

Kasper Adelborg

Nabil Adra

Prasad S. Adusumilli

Ilir Agalliu

Lars Agréus

M. Teresa Agulló-Ortuño

Thomas Ahern

Nihal Ahmad

Atique U. Ahmed

Tim Aitman

Jaffer A. Ajani

R. A. Al-Naggar

Taleb H. Al-Tel

Costantine Albany

Simak Ali

Emma H. Allott

Maria Alsina

Peter Altevogt

Rosario Amato

Srikanth R. Ambati

Peter F. Ambros

Maria R. Ambrosio

Billy Amzal

Sudarshan Anand

Carey K. Anders

Annie Anderson

Kristin E. Anderson

Fabrice André

Nicolas André

Gabriella Andreotti

Zhiwei Ang

Roberto Angioli

Andrea Anichini

Hendrik Jan Ankersmit

Christina M. Annunziata

Alan Anthoney

Michael H. Antoni

Stefan Antonowicz

Annika Antonsson

Mikko Anttonen

Fawzi Aoudjit

Thomas Aparicio

Giuseppe Aprile

Rami I. Aqeilan

D. J. Argyle

Hendrik-Tobias Arkenau

Gregory T. Armstrong

Rudolf Arnold

Sidsel Arnsprang

Grazia Arpino

Nuri Arslan

Banu Arun

Johan Askling

Igor Astsaturov

Kyrgidis Athanassios

M Hammad Ather

Chloe Atreya

Federico N. Aucejo

François Audenet

Leonard Augenlicht

Michael J. Ausserlechner

Adnan Aydiner

S. C. Azoury

Reinhard Büttner

Hideo Baba

Jean-Baptiste Bachet

Rajendra Badwe

Catherine Bagot

David Bajor

Shairaz Baksh

Gustavo Baldassarre

Claudia R. Ball

Cynthia Bamdad

Udai Banerji

Shamila A. Bapat

Rathindranath Baral

Ludovic Barault

Jörg W. Bartsch

Benjamin G. Barwick

Murali D. Bashyam

Boris Bastian

Bristi Basu

Oliver Bathe

Harriet Batista Ferrer

G. David Batty

Michael Baum

Daniel Baumhoer

Alain Beck

Holger M. Becker

Filip Bednar

Miguel Bedolla

Alicia Beeghly-Fadiel

Andrew Beggs

Tanios Bekaii-Saab

Avri Ben-Ze’ev

Sara Benitez Majano

Hayley Bennett

Charlotte Benson

Astrid Berger

Leif Bergkvist

Lothar Bergmann

Johannes Berkhof

Rosa Bernardi

Donald A. Berry

Melissa Bersanelli

Joseph R. Bertino

Francesco Bertoni

Andreas Beutler

Joerg Beyer

Krishnan Bhaskaran

Ramya Bhatia

Yan Bi

Giampaolo Bianchini

Roberto Biffi

Alan Bilsland

Gabriela Bindea

Lynne Bingle

Helgi Birgisson

D. Tim Bishop

Trever G. Bivona

Line Bjørge

Esther P. Black

Sarah P. Blagden

Kerry L. Blanchard

Jean-Yves Blay

Jeremy Blaydes

Maria Blettner

Ruth E. Board

Carla Boccaccio

Stefania Boccia

Alan Boddy

Amy Boddy

Paolo Boffetta

C. Richard Boland

Melissa L. Bondy

Mara Bonelli

Andrea Bonetto

Edith Bonnelye

Thaiz Borin

James A. Borowiec

Josep Maria Borràs

F. T. Bosman

Marie-Josee Boucher

Simon Bouffler

Liam Bourke

Aurélie Bourmaud

Anne Bowcock

Mark Bower

Laura W. Bowers

David J. Bowrey

Mark Boyd

Marc Bracke

Chiara Braconi

Julie A. Bradley

Marie C. Bradley

Patrick T. Bradshaw

Gerard Brady

Michael Braun

John Bridgewater

Louise A. Brinton

Vance Broach

Bruce Brockstein

Alexander S. Brodsky

Mireille J. M. Broeders

Marieke Broekman

Domenyk Brown

Robert Brown

Kristoffer Brudvik

Wolfgang Brueckl

Silvia Brunelli

Nele Brusselaers

Malte Buchholz

Simonetta Buglioni

Ronald M. Bukowski

J. J. Bultema

Mary Ann Burg

Howard A. Burris

Timo Burster

Francesco Bussu

Ronald Busuttil

John Butler

Richard Buus

Natalia Buza

Peter D. Caie

Magdalena Cal

George Calin

Emiliano Calvo

Fernando Calvo

Patricia Juárez Camacho

David Cameron

Ana Paula Campanelli

Esther Jennifer Campbell

Rikki A. Cannioto

Federico Canzian

Jeffrey Q. Cao

Chris R. Cardwell

Mariko Carey

Amancio Carnero

Sara Carpi

Prudence Carr

Judith E. Carroll

Ross Carruthers

Brett W. Carter

Richard Carvajal

Luis G. Carvajal-Carmona

Daniel E. Carvajal-Hausdorf

Oriol Casanovas

Angela Cashell

Javier S. Castresana

David Cella

Giovanni Cerisoli

Byung Joo Chae

Heather J. Chalfin

David Lok Hang Chan

Dorothy Chan

Jefferson Y. Chan

Stephen L. Chan

Dhyan Chandra

David Chang

Helena R. Chang

Robert Chapkin

Benoit Chassaing

Etienne Chatelut

Ian Chau

Ceshi Chen

Fei Chen

Ke Chen

Shiuan Chen

Size Chen

Kwok-Leung Cheung

Johanna Chiche

Fiona Chionh

Gabriela Chiorean

John M. Chirgwin

David D. Chism

Takeshi Chiyomaru

W. J. Chng

Doo Ho Choi

Minsig Choi

Alan Christie

Daniel C. Chung

Vincent Chung

Fortunato Ciardiello

Loredana Cifaldi

Geoffrey J. Clark

Gary M. Clifford

Shamshad Cockcroft

V. Codd

Paul Cohen

Andrew James Coldman

Helen Coleman

A. Dimitrios Colevas

Peter Collins

Daniele Filippo Condorelli

C. Conrad

Zuelma Contreras

Michael B. Cook

Natalie Cook

Colin S. Cooper

Jennifer Anne Cooper

An Cooseman

Mhairi Copland

Erin Cordeiro

Tony Corfield

Beatrice Cormier

Stefanie Corradini

Pippa G. Corrie

Aurélien Corroyer-Dulmont

Laura Cortesi

J. L. Costa

Cedric Coulouarn

Veerle M. H. Coupe

Sarah Coupland

Amanda Coutts

Fiona Cowie

Tracy Crane

Nicola Cresti

Canien Creutzberg

Nicolae Crisan

Greg O. Cron

Deirdre P. Cronin-Fenton

Emma Crosbie

Tom Crosby

Nicholas C. P. Cross

Zoran Culig

Giuseppe Curigliano

Geoff Cuvelier

Jack Cuzick

Brian G. Czito

Begoña Díaz

Maurizio D’Incalci

Toos Daemen

Kiran Dahiya

Maria Dalamaga

Angus G. Dalgleish

Tina Dalianis

Chendil Damodaran

William Damsky

Adam Dangoor

Rachel Dankner

Sarah Danson

Sarah Darby

Benu Brata Das

Francisco Dasi

Adil I. Daud

Sarah E. Daugherty

Elizabeth A. Davies

John R. Davies

Michael A. Davies

Neil Davies

Sarah Davis

Chi-Ping Day

Pradip De

Ugo De Giorgi

David J. De Graff

FR de Gruijl

Jill de Jong

Steven de Jong

Gilles W. De Keulenaer

Inge M. C. M de Kok

Harry J. de Koning

Macarena De La Fuente

Marc de Perrot

Olivier De Wever

Ronald de Wit

Frank Dekker

Robert Dellavalle

Thanh H. Dellinger

Marco Demaria

Shadmehr Demehri

Amanda F. Dempsey

David T. Denhardt

Susan F. Dent

Alexander Deutsch

Nandini Dey

Frédéric Di Fiore

James J. Dignam

QingQing Ding

Luc Yves Dirix

N Utku Dogan

Enric Domingo

Jenny L. Donovan

Frede Donskov

Mehmet Tevfik Dorak

Olivier Dormond

Jonathan E. Dowell

Timothy R. Driscoll

Steven G. DuBois

Anna Dubrovska

Stephen W. Duffy

Laurie Dunn

Francine Durocher

Harold F. Dvorak

Tadeusz Dyba

Martin Dyer

Lars Dyrskjøt Andersen

Julie Earl

Martin M. Eatock

Renee Ebisch

Julien Edeline

Angela B. Edgar

Dylan R. Edwards

Hellen Edwards

Joanne Edwards

Alexey Efanov

Kathleen M. Egan

Lawrence H. Einhorn

Tim Eisen

Imane El-Dika

Bassel F. El-Rayes

K. Miriam Elfström

Peter Elwood

Gerda Englholm

D. Ennishi

Mathew Erhardt

Stefan Erkeland

Christophe Erneux

Alexandre E. Escargueil

Pablo V. Escriba

Ferry A. L. M. Eskens

Alan Evans

D. Gareth Evans

Carl Johan Fürst

Guy B. Faguet

Nicole Fahrenbacher

Benjamin Fairfax

Stefano Fais

Jean Faivre

Sandrine Faivre

Corinne Faivre-Finn

Gerald S. Falchook

Stephen J. Falk

Mohammad Fallahi-Sichani

Marie Fallon

Matteo Fassan

Louise Fearfield

Richard G. Feltbower

Hui Feng

J. Christopher Fenno

Laura Ferraro

Robert Figlin

Christine Fischer

James M. Flanagan

Ian N. Fleming

Olivia Fletcher

Gunnar Folprecht

Francesca Fornari

Lorenzo Fornaro

Lynne Fiona Forrest

Martin Forster

Renée Turzanski Fortner

Paul Alexander Foster

Steven Scott Foster

Dimitrios Fotiadis

William D. Foulkes

Markus Frank

Hester Franks

Callum Fraser

Irma Fredriksson

Michael R. Freeman

M. P. Freire

Rafael Fridman

Claire Friedman

Karlheinz Friedrich

Fieke Froeling

Shen Fu

Marc-Aurel Fuchs

Ezequiel M. Fuentes-Panana

Chunkit Fung

Suzanne A. W. Fuqua

Marike Gabrielson

Timo Gaiser

Umberto Galderisi

Vijaya L. Galic

Gary E. Gallick

Eve Gallop-Evans

Deborah L. Galson

Sofia R. Gameiro

Trivadi S. Ganesan

Daming Gao

Ling Gao

Alex Gaorgakilas

Xavier Garcia del Muro

George Garinis

Elizabeth Garrett-Mayer

Robert A. Gatenby

Aditya Gaur

Jeanine Genkinger

David E. Gerber

Marco Gerlinger

David A. Gewirtz

Milan S. Geybels

T. A. Gheita

Francois Ghiringhelli

Matteo Giaj Levra

Gianluigi Giannelli

Don L. Gibbons

Geoffrey Gibney

Ziv Gil

Duncan Charles Gilbert

Scott Gilbert

Roopinder Gillmore

Pelosi Giuseppe

Martin E. Gleave

Paul Glen

Bengt Glimelius

Ajay Goel

Thorsten Goetze

David E. Goldgar

Simon W. Gollins

Andrea Gombos

Zhihong Gong

Nancy B. Gordon

Kylie Gorringe

Margot A. Gosney

Heike I. Grabsch

Janet Graham

Steven Grant

Mel Greaves

Mark H. Greene

Diana Greenfield

Alastair Greystoke

Giovanni Grignani

John D. Groarke

Jean-Jacques Grob

Irina Gromova

Patti Groome

Guowei Gu

Henk-Jan Guchelaar

Hugo Guerrero-Cázares

Rachel Guest

William J. Gullick

Jayarama B. Gunaje

Venugopal Gunda

Emma Guns

Jianping Guo

Ashish Gupta

Sanjay Gupta

Shlok Gupta

Ana Gutiérrez-Fernández

Geertruida H. de Bock

Angela Hague

Jorg Haier

Gunnel Hallden

Omid Hamid

William T. Hamilton

Pascal Hammel

J. W. Han

Jiali Han

Andrew M. Hanby

Mamoru Harada

James J. Harding

Adrian L. Harris

Ewen Harrison

Andrew Robert Hart

Arndt Hartmann

Mathieu Hatt

Hege Sagstuen Haugnes

Jari Haukka

Mickael Hauptman

Maria A. Hawkins

Yoku Hayakawa

Shin-ichi Hayashi

Philip Haycock

John D. Hayes

Nicholas Hayward

Wei He

Christopher M. Heaphy

David F. Heigener

Eveline A. M. Heijnsdijk

Lucie Heinzerling

David M. Helfman

Martina Heller

Samantha Hendren

Jolyon Hendry

Christopher Hewitt

Maria Hewitt

Mats Heyman

Dominique Heymann

Cinta Hierro

Allan Hildesheim

Mark Hill

Mohan Hingorani

Yasemin Hirst

Henrik Hjalgrim

Matthew Hoare

Thomas Hoehler

Jonathan N. Hofmann

Stefan Holdenrieder

Antoine Hollebecque

Jeff Holly

Neil S. Horowitz

Dirk Hose

David W. Hoskin

Yariv Houvras

Alexandra C. Hristov

Cheng-Lung Hsu

Cai Huang

Qi-tao Huang

Ruby Y. J. Huang

Xin Huang

Richard Hubner

Meritxell Huch

Kevin S. Hughes

Florence Huguet

Rayjean J. Hung

David G. Huntsman

Katherine E. Hutchinson

Jin Won Hyun

Torukiri I. Ibiebele

Masafumi Ikeda

David H. Ilson

Mohammad Ilyas

Stefan Indraccolo

Manami Inoue

Heikki Irjala

Sofie Isebaert

Yuichi Ishikawa

Gustavo Ismael

Motoki Iwasaki

Graham H. Jackson

Jamie Jacobs

Anders Jakobsen

Shadia Jalal

Nigel B. Jamieson

Wolfgang Janni

Tobias Janowitz

Henry Jensen

Lars Henrik Jensen

Jae-Wook Jeong

Carmen Jeronimo

Claire F. Jessup

Long R. Jiao

Renjie Jin

Heikki Joensuu

Christoffer Johansen

Peter W. M. Johnson

Philip J. Johnson

Christopher Jones

Lee W. Jones

Rena Jones

Robin L. Jones

Claus Jorgensen

Ian Judson

Klaus Jung

Manfred Jung

Bruno Köhler

Humam Kadara

Kaoru Kahata

Ersan Kalay

Bozena Kaminska

Priyanka Kanth

Tatsuya Kanto

Premashis Kar

Niki Karachaliou

Margaret R. Karagas

Amalia Karahalios

Giorgos Karakousis

Eva Karamitopoulou

Per Karlsson

R. Jeffrey Karnes

Florian Karreth

Ioannis Karydis

Pashtoon M Kasi

Bernd Kasper

Susan Kasper

Karin Kast

Efstathios Kastritis

Alexander Katalinic

Jakob Kather

Masaru Katoh

Elad Katz

Ai-Wu Ke

Bhumsuk Keam

Linda E. Kelemen

Charles Kelly

Scott P. Kelly

Nancy E. Kemeny

Paul M. Kent

Robert Stephen Kerrison

Dineo Khabele

J. Khales

Chan Kyo Kim

Dokyoon Kim

Nam Kyu Kim

Richard Kim

Young Tae Kim

Leo J. Kinlen

Ben Kinnersley

Nicole K. Kiss

Yasuhide Kitagawa

Alexander Kleger

Petra Klose

Jan Klozar

Jeffrey Knauf

Jennifer Knox

Ben C. B. Ko

Aung Ko Win

Lindsay C. Kobayashi

Meri Koivusalo

Hanna Kokko

Shuhei Komatsu

Naru Kondo

Anthony Kong

Xiangrong Kong

Dong Hoe Koo

Sittichai Koontongkaew

Scott Kopetz

Martin Koskas

Ilona Kovalszky

Christoph Kowalski

Clemens Krepler

Malathi Krishnamurthy

Roland P. Kuiper

Addanki P. Kumar

Deepak Kumar

Rajiv Kumar

Satish Kumar

Shaji Kumar

Ken-ichi Kusakabe

Natasha Kyprianou

Maria-Christina Kyrtsonis

Derek Kyte

Heinz Läubli

Myriam Labelle

Nicholas Lakin

Aly-Khan A. Lalani

Tram Kim Lam

Angela Lamarca

Paul Lambert

Samy Lamouille

Maria Grazia Lampugnani

Stephanie R. Land

Ola Landgren

Terry H. Landowski

T. Lange

James M. Larkin

Alessandra Larocca

Signe Benzon Larsen

Peder Larson

L. Larue

Pierre Laurent-Puig

Sean E. Lawler

Claudia Helga Dorothee Le Guin

Émilie Le Rhun

Christophe Le Tourneau

Franck Lebrin

Christy J. W. Ledford

Hyun-Shik Lee

Mei-Hsuan Lee

Victor H. F. Lee

Stefan Leger

Natasha Leighl

Anthony Lemarie

Hannah Lennon

Pauline Leonard

Zohar Levi

Douglas A. Levine

Brian Lewis

John Lewis

Cheng-Chieh Li

Fengzhi Li

Jinjun Li

Nan Li

Shau-Hsuan Li

Shulin Li

Tsai-Chung Li

Wenliang Li

Xia Li

Xin Li

Yong Li

Vasilios Liapis

Arthur Liberzon

Cornelia Liedtke

Triantafillos Liloglou

Annette Lim

Darren Lim

Wan-Teck Lim

Y. Lin

Corinne M. Linardic

Claus Lindbjerg Andersen

Annika Lindblom

Daniel J. Lindner

Colin R. Lindsay

Martha Linet

Michael Linnebacher

Lance Liotta

Kevin Litchfield

Ai-Qun Liu

Fei-Fei Liu

Stanley K. Liu

Xiaoman Liu

Zheng-Gang Liu

Lorenzo Livi

Paul Loadman

Fiona Lofts

Anna E. Lokshin

Martijn Lolkema

Niklas Loman

Juanita Lopez

Luis A. Lopez-Fernandez

Christopher J. Lord

Simon Lord

Paul Lorigan

Maeve A. Lowery

Chang Hsien Lu

Zhen Lu

Marie Ludvikova

Alessandro Lugli

A. Lugo

Jun Luo

Philip Lupo

Brigid M. Lynch

Conor Lynch

Georgios Lyratzopoulos

Johan Lyth

Markus Möhler

Henrik Møller

Yuk Ting Ma

Monique Maas

M. Macías-González

Valentine M. Macaulay

William J. Mackillop

Iain R. J. Macpherson

Robert M. Mader

Ayman Madi

Devalingam Mahalingam

K. K. Mahato

Jane Maher

Stephen G. Maher

Sabine Mai

Michael L. Maitland

M. Makishima

Umberto Malapelle

Frédérick Malette

Hassan Z. Malik

Judith A. Malmgren

Bernard Manger

Sridhar Mani

Judith Manola

Zhiyong Mao

Victoria J. Mar

Anthony Maraveyas

Paola Marcato

Federica Marchesi

Francesca Marcon

M. Mareel

Kim A. Margolin

Camille Maringe

Maurie Markman

Sharon Marsh

Ernest Marshall

John W. M. Martens

Elena Martens

Alberto Martin

Miguel Martin

Patricia Martin-Romano

Ana Carolina Martinez Torrez

Chiara Martinoli

Sandra F. Martins

Michal Masarik

Luca Mascitelli

Nader N. Massarweh

Gregory A. Masters

Marie Mathers

Andres Matoso

Koji Matsuo

Patrick Matthias

Melissa Matz

Tim S. Maughan

Anne M. May

Astrid Mayer

Lindsey D. Mayo

Guillermo Mazzolini

Jessica N. McAlpine

Caroline E. McCoach

John McGregor

W. P. McGuire

Colin McKay

Úna McMenamin

Donald C. McMillan

Mairead Geraldine McNamara

Janice M. Mehnert

Alexander Melamed

Gerry Melino

Anders Mellemgaard

Usha Menon

Franco Merletti

Sue Merrick

Malek Meskawi

Wilma E. Mesker

Christina Messiou

Tim Meyer

Gabor Mezei

Kyriaki Michailidou

Judith Michels

Caroline Michie

Jaap M. Middeldorp

Paolo Mignatti

Anne Miles

Koshi Mimori

Olivier Mir

Alexander Mirnezami

Shiraz I. Mishra

Elizabeth D. Mitchell

Anirban K. Mitra

Monika Mitra

Sumegha Mitra

Ingvil Mjaaland

Simone Mocellin

Khalid S. Mohammad

Damian J. Mole

David R. Mole

Olivier Molinier

Alfredo Molinolo

Silvio Monfardini

Guy H. Montgomery

Anthony V. Moorman

Andreas Moosmann

Gil Mor

Kelley W. Moremen

Victor Moreno

Carys Morgan

Maria P. Morgan

Masaki Mori

Richard Moriggl

Hamid Morjani

Markus Morkel

Andrea Morrione

David Morris

Eva Judith Ann Morris

Van Morris

Cynthia C. Morton

Jennifer Morton

Lindsay M. Morton

Maria Moschovi

Susan J. Moug

Aristidis Moustakas

Carmen Muñoz

Claudius Mueller

Colin R. Muirhead

Bhramar Mukherjee

Somnath Mukherjee

Deborah Mukherji

Asok Mukhopadhyay

Daniel Munoz Espin

Pamela N. Munster

Peter Murchie

Gwen Murphy

Brion W. Murray

Graeme I. Murray

Paola Muti

Sylvie Négrier

Jean-Marc Nabholtz

N. Nadiminty

Y. Nakabeppu

Keiichiro Nakamura

Tomoki Nakamura

Yusuke Nakamura

Joo-Hyun Nam

Vikneswaran Namasivayama

Margherita Nannini

Steven A. Narod

Kent Nastiuk

Rachael Natrajan

Jean-Charles Nault

Eva Negri

Erik Nelson

Kenneth P. Nephew

Kirsten Ness

Romana T. Netea-Maier

Cindy Neuzillet

Patrick Neven

Kimmie Ng

Samuel Ngan

Shin Foong Ngiow

Iain D. Nicholl

Nils H. Nicolay

Daotai Nie

Anders Lade Nielsen

Hans Nijman

Aleksandra Nikolić

Maja Niksic

Mef Nilbert

Reiko Nishihara

Ulrich Nitsche

Njiro Nohata

Katsuhiko Nosho

Fiona Nussey

Karin Nylander

Lennarth Nystrom

James P. B. O’Connor

Jake O’Donnell

Peter H. O’Donnell

Peter O’Dwyer

Tracy O’Mara

Eric O’Neill

Derek O’Reilly

Daniel O. Stram

Takahiro Ochiya

Karin Oechsle

Judith Offman

Shuji Ogino

Hannah Oh

Akinyemi Ojesina

Cem Onal

Jason Ong

Sheina Orbell

Nobuhiko Oridate

Giulia Orlando

Tomo Osako

Lobna Ouldamer

Philip Owens

Beatriz Pérez-Gómez

David Page

Marina Pajic

Claudia Palena

Giuseppe Palmieri

G. E. Pamuk

Hongchao Pan

Shin-Liang Pan

Klaus Pantel

Salvatore Papa

Gianpaolo Papaccio

Demetris Papamichael

Angelo Virgilio Paradiso

Emilio Parisini

Boyoung Park

Jin Young Park

Pyong Park

So Yeon Park

Christine L. Parr

Christopher N. Parris

Jason L. Parsons

Harvey I. Pass

Alpa Patel

Rakesh P. Patel

Depananda Pati

P. Patrignani

Jim Paul

Sonya Pavlovic

Graham Pawalec

Charlotte Pawlyn

Semra Paydas

Miranda Payne

Julien Péron

Fedro A. Peccatori

Donna Peehl

Vicente Peg

Frank Peinemann

Melike Pekmezci

Paivi Peltomaki

Trevor Penning

George Pentheroudakis

Aurora Perez-Cornago

Sandra Perez-Torras

Claire M. Perks

Augusto Pessina

Christopher J. Peters

Jessica L. Petrick

Anreas Pettersson

Russell D. Petty

Francesco Pezzella

James M. Phang

Paul D. Pharoah

Xuan-Anh Phi

Roger M. Phillips

Filippo Pietrantonio

Camilla Pilati

Paul F. Pinsky

Luigi Pirtoli

Xavier Pivot

Mary C. Playdon

Ruben R. Plentz

Sharon Plon

Seth Pollack

Giuseppe Portella

David Porter

Sophie Postel-Vinay

Nicholas Pote

Chantevy Pou

Poulikos Poulikakos

Mohamad Amin Pourhoseingholi

Carla Prado

Rajendra Prasad

Aleix Prat

Christine Pratt

Thomas Prebet

Timothy J. Price

Charlotte Proby

Maria Pia Protti

Elena Provenzano

Sylvain Provot

Amanda Psyrri

Mark P. Purdue

Rituraj Purohit

Martine Puts

Haili Qian

Claus Rödel

MA Røder

Antonio M. Rabasco-Álvarez

Derek C. Radisky

Elizabeth Raetz

Derek Raghavan

Lola Rahib

P. Rajaraman

Suresh S. Ramalingam

Shankar Raman

Ramesh K. Ramanathan

Rebeca Ramis

Sheela Rao

George Zacharias Rassidakis

Nancy Ratner

Yaddanapudi Ravindranath

Olga V. Razorenova

Neal E. Ready

Francisco X. Real

Matejka Rebolj

Sangeetha Reddy

H. Paul Redmond

Malcolm Ronald Walter Reed

Helen Louise Reeves

Tarik Regad

Jorge S. Reis-Filho

Christoph Reissfelder

Cristina Renzi

David F. Restuccia

Edyta Reszka

Raoul C. Reulen

Chris Reutelingsperger

Rodolfo Rey

Christine Reynaert

Andrew R. Reynolds

W. J. Rhee

T. L. Rižner

Jeremy N. Rich

Brent Richards

Paul G. Richardson

Paul Ridgway

Alistair E. Ring

Anupam Rishi

Debra Ritzwoller

Kathryn Robb

Jacques Robert

Martha Robles-Flores

Andrew W. Roddam

Francis Rodier

Rene Rodriguez

Cristina Rodriguez-Antona

Sabine Rohrmann

Christian Rolfo

Isabelle Romeiu

Dana M. Roque

Rafael Rosell

Gary Rosner

Paul J. Ross

Antonio Rossi

Campbell S. D. Roxburgh

Patricia Roxburgh

Charles Rubin

Greg Rubin

Marek Ruchala

Kathryn J. Ruddy

Nandini Rudra-Ganguly

Tatyana Ruksha

Simon Rule

Antonio Russo

Gordon J. S. Rustin

Mark J. Rutherford

Steve Ryder

Joseph Sacco

Adrian G. Sacher

Rosa M. Sainz

Hiroshi Saito

Kazutaka Saito

George H. Sakorafas

Gianluca Sala

Lucas A. Salas

Ramon Salazar

David S. Salomon

Leslie Samuel

Milena Sant

Daniele Santini

Sofia Nascimento Santos

Diana Sarfati

Bruno Sarmento

Andrea Sartore-Bianchi

Sven Saussez

Edward A. Sausville

Elias J. Sayour

Joachim Schüz

T. J. Schall

Howard I. Scher

Marielle Scherrer-Crosbie

Michael E. Scheurer

Joellen M. Schildkraut

Marianne Schmid

Clemens Schmitt

Günter Schneider

John Schofield

Anna Schuh

Judith Ann Schwartzbaum

Heidi Schwarzenbach

Andreas Scorilas

Aaron Scott

David Scott

Michael J. Seckl

Christoph Seidel

Sanjeewa Seneviratne

Carlo Senore

Salvatore Serravalle

Arun Seth

Riyaz Shah

Priyanka Sharma

Rohini Sharma

Heather M. Shaw

Gemma Shay

Feng Shen

Hongbing Shen

Trevor G. Shepherd

Qian Shi

Wenyin Shi

Xianglin Shi

Kohei Shigeta

Xianglin Shih

Daniel Shin

Hitoshi Shiozaki

Alexander Noor Shoushtari

Emma Shtivelman

Ke Shuai

Kris Ann Shultz

Lu Si

Frank Sicheri

David Sidransky

Peter M. Siegel

Ines Silva

Andrew Silver

R. H. Silverman

Michael Simon

Rodney Sinclair

Amar B. Singh

Marianne Sinn

Daniel Sinnett

Carole Siret

Freddy Sitas

Raquel Sitcheran

Ka Tat Siu

Tobis Sjoblom

Daina Skiriutė

Nicholas Skuli

Patrick M. A. Sleiman

Cristian Smerdou

Vincent T. H. B. M. Smit

Samuel George Smith

Elizabeth C. Smyth

Petur Snaebjornsson

Alex Snyder

Carl H. Snyderman

Robert W. Sobol

Davendra P. S. Sohal

Huan Song

Jie-Young Song

Montse Soriano-Gabarró

Andrea Sottoriva

Pavel Soucek

Hugo Sousa

Nuno Sousa

Paul N. Span

David Spiegel

Daniel E. Spratt

Amanda Spurdle

Adrian Stanley

Giorgio Stanta

Martin Stanulla

Naureen Starling

Justin Stebbing

Nicola Steele

Rob Stein

Alexander Steinle

Arnulf Stenzel

Albrecht Stenzinger

William G. Stetler-Stevenson

Jon Stobo

Peter Storz

Björn Strander

Euan Stronach

Amit Sud

Hiromi Sugiyama

Frank Sullivan

Ryan Sullivan

Farhana Sultana

Shi-Yong Sun

Yu Sun

Arthur Sun Myint

M. R. Sussman

Calum D. Sutherland

Jaim Sutton

John Sweetenham

Stefan Nicholas Symeonides

Ildiko Szabo

Jacek Tabarkiewicz

Takanobu Tagawa

Alphonse G. Taghian

Diana Tait

Stephen Tait

Jukka Takala

Shigetsugu Takano

Shinsuke Takeno

Gesche Tallen

Rulla M. Tamimi

Ming Tan

Masamitsu Tanaka

Chad Tang

Cullen Taniguchi

Nizar Tannir

Ian F. Tannock

Woan-Yuh Tarn

Georgi Tchernev

Rajeshwar Tekmal

Achim Temme

Lauren R. Teras

Masanori Terashima

Howard Terebelo

Vinay Tergaonkar

Amanda Termuhlen

Kathryn L. Terry

Krishnansu S. Tewari

H. H. Thein

Christina Therkildsen

Alain R. Thierry

Stephen Thompson

Fiona Thomson

John Thomson

Mangesh Thorat

De-an Tian

Madeleine M. A. Tilanus-Linthorst

Nahira Timilshina

John F. Timms

Paul Timpson

Debu Tipathy

Jasmin A. Tiro

Vivianne C. G. Tjan-Heijnen

Sara Michell Tolaney

Ian P. M. Tomlinson

Adetunji T. Toriola

Tibor Tot

Yann Touchefeu

Lara Traeger

Lois B. Travis

Anouk Kirsten Trip

Giancarlo Troncone

Claudia Trudel-Fitzgerald

Nicolas Tsavaris

Lap-Ah Tse

Clare Turnbull

James Turvill

Tove F. Tvedskov

Christina Twyman-Saint Victor

Scott Tyldesley

Hiroji Uemura

Rainy Umbas

Takashi Umemura

Meena Upadhyaya

Katsuhiro Uzawa

Celine M. Vachon

Ratna K. Vadlamudi

Claire M. Vajdic

Edith Valdez-Martinez

Gautam Kishore Valecha

Nicola Valeri

Juan W. Valle

Bart van de Sluis

Cornelis J. H. van de Velde

Desiree van den Bongard

Jan Van den Bossche

Flora E. van Leeuwen

Sandra Van Schaeybroeck

Isabelle Van Seuningen

Brian A. Van Tine

Jonna van Vulpen

Hanna van Waart

Udo Vanhoefer

Guillaume Vares

Sanjeev Vasudevan

Chittibabu Vatte

Ines Vaz-Luis

Mary Anna Venneri

Francisco E. Vera-Badillo

Marcel Verheij

Helena M. Verkooijen

James Vestey

Silvestre Vicent

Sergi Vidal-Sicart

Mark Vincent

Marco Vinceti

Ilio Vitale

Arndt Vogel

Christian von Buchwald

Stephan von Haehling

Erik von Seth

Paresh Vyas

Graham W. Warren

Nic Waddell

Jon Wadsley

Nolan A. Wages

Lars M. Wagner

Richard Wakeford

Michael Wakelam

Heather A. Wakelee

Lucy Wall

Heather M. Wallace

Zachary Wallace

Kyle M. Walsh

Tom Walsh

Fiona M. Walter

Jianli Wang

Peizhong Peter Wang

Qiming Jane Wang

Weiguang Wang

Douglas G. Ward

Harpreet Wasan

Masayuki Watanabe

Justin Waters

John Charles Waterton

David Watkins

R. William

G. Watson

T. Watts

Penelope M. Webb

Georg F. Weber

Ashani T. Weeraratna

Andrew Wei

Neils Weinhold

Piri Welcsh

Ulrich Wellner

Paul Welsh

Catharine M. L. West

Kenneth Westover

Paul Wheatley-Price

Beck White

Emily White

Jeff D. White

Armin Wiegering

Joseph Wiemels

Hans Wildiers

Anna Clare Wilkins

Holger Willenberg

Walter Willett

John A. Williams

Anna-Lise Williamson

Stuart Charles Williamson

William R. Wilson

D. M. Winn

Des Winter

Kerri M. Winters-Stone

Penella J. Woll

C. C. Wong

Stephen Q. Wong

Cameron Wright

Woodring Wright

Qijun Wu

Xiaohua Wu

Yihua Wu

W. Wulaningsih

Wahyu Wulaningsih

Lynda Wyld

Wei Xiao

Shao-An Xue

Lusine Yaghjyan

Krishna Yalla

Tesshi Yamada

Masayuki Yamamoto

Motohisa Yamamoto

Noboru Yamamoto

Katsuhiko Yanaga

Chih-Hsin Yang

Timothy A. Yap

Linda S. Yasui

Lucy Yates

John Yaxley

Tsz Lun Yeung

Min Yi

Howard Yim

Zhang Yingjian

Desmond Yip

Kazuhiro Yoshida

Caroline Young

Paul W. Young

George M. Yousef

Kenneth H. Yu

Ying Yuan

Mathew Yurgelun

Nadia Zaffaroni

Nousheen Zaidi

F. Zalazar

John Zalcberg

Khalil Zaman

Rita Zamarchi

Xingxing Zang

A. G. Zeimet

Yoh Zen

Jincheng Zeng

Shan Zeng

Donna Zhang

Jinfeng Zhang

Li Zhang

Shawn (Xiang) Zhang

Shutian Zhang

Xiaodong Zhang

Xiaowen Zhang

Xinhua Zhang

Yi Zhang

Zheng-Yun Zhang

Zhiqian Zhang

Zuo-Feng Zhang

Peiming Zheng

Dawang Zhou

Guanglei Zhuang

Alessandra Zingoni

Zhao Zuowei

